# A Case Report of Crotalidae Immune F(ab’)_2_-associated Coagulopathy Recurrence in a Preschool-age Child

**DOI:** 10.5811/cpcem.6583

**Published:** 2024-05-14

**Authors:** Jean C.Y. Lo, E. Lea Walters, Brian Wolk

**Affiliations:** Loma Linda University Medical Center, Department of Emergency Medicine, Loma Linda, California

**Keywords:** *pediatric*, *case report*, *recurrence*, *F(ab’)*_*2*_

## Abstract

**Introduction:**

Pit viper envenomation may cause coagulopathy. The coagulopathy has been treated with crotalidae polyvalent immune fragment antigen-binding (Fab) ovine antivenom for the last few decades in the United States and usually corrects the acute coagulopathy within hours. Days after receiving Fab, coagulopathy may recur in approximately half of the patients. Another divalent antivenom, crotalidae immune F(ab’)_2_ (equine)–F(ab’)_2_–was approved by the US Food and Drug Administration for the treatment of pit viper envenomation. F(ab’)_2_ is composed of two linked antigen-binding fragments of immunoglobulin G. Several studies have demonstrated that F(ab’)_2_ is less likely to be associated with recurrence. There is no reported case of F(ab’)_2_-associated late coagulopathy in very young patients. We report the first case of recurrence associated with F(ab’)_2_ use in a preschool-age child.

**Case Report:**

A preschool-age male developed leg swelling and hypofibrinogenemia after rattlesnake envenomation. F(ab’)_2_ was administered to stabilize the leg edema and to correct the hypofibrinogenemia. The patient improved clinically and was discharged on hospital day five. Seven days after the rattlesnake envenomation, he returned to the emergency department as instructed. Laboratory data revealed recurrent hypofibrinogenemia.

**Conclusion:**

There are two antivenoms available in the US to treat crotalid envenomation, Fab and F(ab’)_2_. F(ab’)_2_ is less likely to be associated with recurrent coagulopathy in comparison to Fab. We report the first case of recurrence associated with F(ab’)_2_ in a preschool-age child. It is important that the emergency physician be aware of potential F(ab’)_2_-associated recurrent coagulopathy. Adult and pediatric patients may need to follow up to be evaluated for hypofibrinogenemia and/or thrombocytopenia after receiving F(ab’)_2_.

Population Health Research Capsule What do we already know about this clinical entity?

*Recurrent coagulopathy occurs commonly in patients who receive Fab. Recurrence is less common in patients who received crotalidae immune F(ab’)_2_ (equine)–F(ab’)_2_.*

What makes this presentation of disease reportable?

*Until now there has been no reported case of recurrence in very young patients who received F(ab’)_2_. Our patient developed hypofibrinogenemia one week after initial treatment.*

What is the major learning point?

*Recurrence of hypofibrinogenemia may occur in patients of all ages who receive F(ab’)_2_ after rattlesnake envenomation.*

How might this improve emergency medicine practice?

*Emergency physicians who administer F(ab’)_2_ to patients after rattlesnake envenomation should recommend close follow-up in a week.*

## INTRODUCTION

Pit viper (*Crotalinae*) envenomation in North America may cause coagulopathy, which manifests as hypofibrinogenemia and thrombocytopenia.^1^ The coagulopathy has been treated with crotalidae polyvalent immune Fab (ovine) (Fab) for the last few decades in the United States.^2^ While Fab corrects the initial coagulopathy within hours, days after initial Fab administration, coagulopathy may recur.^1^ ^,^ ^3^ ^,^ ^4^ ^,^ ^5^ Recurrent coagulopathy may be due to rapid clearance of Fab, which has an effective half life of less than 12 hours.^1^ Approximately half of the patients who received Fab develop recurrent hypofibrinogenemia or thrombocytopenia.^1^ The concern with recurrence phenomenon is increased risk of bleeding.^1^ ^,^ ^3^ ^,^ ^4^

Another antivenom, crotalidae immune F(ab’)_2_ (equine)–F(ab’)_2_–was approved by the US Food and Drug Administration (FDA) in 2015 for the treatment of North American pit viper envenomation.^6^ F(ab’)_2_ is composed of two linked fragment antigen-binding regions with a longer half-life than Fab.^7^ Several studies have demonstrated that F(ab’)_2_ is less likely to be associated with recurrence.^7^ ^,^ ^8^ ^,^ ^9^ Previous reported cases of F(ab’)_2_-associated recurrence were noted in adults and children >10 years old. We report the first case of recurrent coagulopathy associated with F(ab’)_2_ in a preschool-age child.

## CASE REPORT

A preschool-age male presented to an outside emergency department (ED) after rattlesnake envenomation to his leg. F(ab’)_2_ 10 vials were given at the outside hospital prior to transfer to a pediatric ED. Vital signs revealed temperature 98.1^o^ Fahrenheit, blood pressure 105/64 millimeters of mercury, heart rate 108 beats per minute, respiratory rate 18 breaths per minute, and oxygen saturation 97%. Physical exam demonstrated left leg edema with ecchymosis. The initial fibrinogen was 178 milligrams per deciliter (mg/dL) (reference range 200–393 mg/dL). The patient developed worsening leg swelling. He received F(ab’)_2_ 10 vials in the ED and was admitted to the pediatric intensive care unit (PICU). An additional 28 vials of F(ab’)_2_ were given in the PICU (48 vials in total). His leg edema and ecchymosis stabilized. No other source of bleeding was noted. His fibrinogen and platelet level remained within normal range during the admission. The patient was discharged on day five.

He returned to the ED seven days after envenomation. The ED evaluation revealed fibrinogen of 147mg/dL. Subsequent ED visit three days later revealed fibrinogen improved to 162 mg/dL. The Table includes his fibrinogen trend after rattlesnake envenomation. His leg edema and ecchymosis improved. No thrombocytopenia was noted during admission or follow-up.

**Table.** Fibrinogen trend between day one and day 10 after rattlesnake envenomation.

Day after rattlesnake envenomation	1	2	3	4	7	10
Fibrinogen level milligrams per deciliter (reference range 200–393)	176	396	283	297	147	162

## DISCUSSION

Approximately 5,000 snake envenomations are reported to poison control centers each year in the US.^10^ The majority of these snake envenomations are secondary to rattlesnake envenomation.^11^ *Crotalinae* envenomation is predominately associated with local tissue effect and hematologic effects. Hematologic effects are treated with antivenom. Over the last few decades, Fab has been the primary treatment for *Crotalinae* envenomation in the US.^2^ The platelet level and fibrinogen level typically improve within hours of antivenom administration. In approximately half of the patients the platelet and/or fibrinogen levels drop again a few days after initial Fab administration.^1^ Patients are instructed to follow up with a primary care physician or return to the ED to recheck their platelet and fibrinogen levels a few days after initial Fab administration.

Patients who develop recurrence require close follow-up with frequent measurement of platelet and fibrinogen levels. Severe thrombocytopenia or hypofibrinogenemia have been associated with increased risk of bleeding.^4^ ^,^ ^5^ ^,^ ^6^ Another antivenom F(ab’)_2_ was approved by the FDA in 2015.^6^ F(ab’)_2_ is larger in size in comparison to the Fab fragment and is thought to have a slower renal clearance and longer half-life. The benefit of F(ab’)_2_ over Fab is that F(ab’)_2_ is less likely to result in recurrence due to longer half-life.

Now there are two competing antivenoms. F(ab’)_2_ appears to have a lower risk of recurrent coagulopathy in multiple studies. A randomized-controlled trial by Bush et al that compared Fab and F(ab’)_2_ revealed that F(ab’)_2_ is less likely to be associated with recurrence phenomenon. While 29.7% of patients in the Fab group developed recurrence, 10.3% patients in the F(ab’)_2_ group developed recurrence. The platelet counts and fibrinogen levels were lower in the Fab group than those in the F(ab’)_2_ group. All six patients who developed recurrence after receiving F(ab’)_2_ lived in inland Southern California. One possibility is that rattlesnake envenomation in inland Southern California is more likely to be associated with late coagulopathy. Furthermore, the study by Bush included 29.3% of patients in the F(ab’)_2_ group who were younger than 10 years old. None of the children <10 years developed recurrence.^7^

A study by Mascarenas et al also reported no late coagulopathy associated with F(ab’)_2_. Mascarenas compared 37 patients, 11 in the F(ab’)_2_ group and 26 in the Fab group. The rate of coagulopathy was 0% in the F(ab’)_2_ group and 29% in the Fab group.^8^ A study by Boyer et al demonstrated that more patients who received Fab developed thrombocytopenia and hypofibrinogenemia in comparison to patients who received F(ab’)_2_. Of the six patients who received F(ab’)_2_, one patient developed thrombocytopenia and zero patients had a low fibrinogen level (<150 mg/dL).^9^ These studies demonstrated that recurrence is less likely in patients who received F(ab’)_2_. However, there is still a non-zero risk for recurrent coagulopathy, as illustrated by this case. To date, there has been no reported case of recurrence associated with F(ab’)_2_ use in children <10. We report the first case of F(ab’)_2_-associated recurrence in a preschool-age child without known pre-existing hematopathologic condition.

Limitations of our report include risk of extrapolation from a single case as well as the fact that the fibrinogen concentration did not reach a level that would suggest a high risk of bleeding.^12^ A larger dataset would be required to establish whether laboratory surveillance is mandatory after discharge for children receiving F(ab’)_2_ antivenom.

## CONCLUSION

The antivenom crotalidae immune F(ab’)_2_ (equine) is less likely than Fab to result in recurrence or severe bleeding associated with late coagulopathy. The benefit of F(ab’)_2_ is lower bleeding risk and potentially less frequent follow-up for laboratory testing. While F(ab’)_2_-associated late coagulopathy is infrequent, it does occur. Adult and pediatric patients may still require follow-up for detection of recurrent thrombocytopenia and coagulopathy after receiving F(ab’)_2_.
